# Progress in Obstetrics and Gynæcology

**Published:** 1874-10

**Authors:** A. Reeves Jackson

**Affiliations:** Lecturer on Diseases of Women, Rush Medical College, Chicago


ARTICLE I.—*Progress in Obstetrics and Gynæcology.* By A. REEVES JACKSON, M.D., Lecturer on Diseases of Women, Rush Medical College, Chicago.

1. On the Treatment of Chronic Vaginitis. By Dr. HARRISON. (*Obstetrical Jour. of Great Britain and Ireland*, July, 1874; *Virginia Medical Monthly*, July, 1874.)

2. The Treatment of Amenorrhœa by Galvanization of the Endometrium. By Dr. JAMES J. WHITTAKER, (*The Clinic*, July 25, 1874.)

3. Cauterization of the Uterus. By Dr. WM. A. GILLESPIE. (*Virginia Medical Monthly*, August, 1874.)

4. Chloral Hydrate as an Anæsthetic During Labor. By U. S. PLAYFAIR, M.D. (*London Lancet*, Feb. 21, 1874.)

5. The Normal Temperature, Pulse and Respiration of Puerperal Women. By Dr. G. W. LINN. (*Philadelphia Medical Times*, May 9, 1874.)

1. Dr. Harrison speaks in terms of praise of the following method of treating chronic vaginitis: In obstinate cases, and especially where the touch reveals a thickened and roughened condition of the vaginal walls, it is idle to waste time with weak solutions of alum, sulphate of zinc, nitrate of silver, tannin suppositories, etc. We must proceed at once to the use of the stronger caustics—either chromic acid or the solid nitrate of silver. If chromic acid is used, it should be diluted with an equal quantity of water, and the walls of the vagina having been exposed by a speculum, a thorough application should be made to the diseased surface.* A piece of cotton, saturated with glycerine, having a string attached to facilitate its removal, is then introduced into the vagina, and should be removed by the patient the next morning. Copious injections of flaxseed tea, at a temperature of 98° F., should be used two or three times daily. Hot water injections should also be used morning and evening. The nitrate of silver is preferably used in stick. It causes a good deal of pain for two or three hours; wnen excessive, a solution of common salt must be thrown into the vagina. A glycerine dressing should be introduced after its use, as after the chromic acid.

After the immediate effects of the cauterization have disappeared —and in the case of chromic acid it may require a week—one of Sims' glass dilators, such as he uses after his operation for vaginismus, is introduced, and the patient directed to wear it continuously. It should be dipped in warm water and then in glycerine, before being introduced. In obstinate cases, simple cauterization will disappoint our expectations, but if succeeded by the use of the glass dilator, the worst cases will yield sooner or later; unless, indeed, the disease depend upon incurable constitutional affections.

The favorable action of the dilator is threefold. In the first place, by keeping the inflamed walls of the vagina apart, rest is secured; secondly, with the distension of the vaginal walls, compression is exercised upon their blood vessels, and hyperæmia is moderated; and, thirdly, the pressure upon the hyper-plastic tissue

* [The application of chromic acid, nitrate of silver, or, indeed, any caustic, to an inflamed mucous surface, will be found much more efficient if the part be previously thoroughly cleansed with a solution of chloride of sodium, followed with warm water. J.]

causes its atrophy. The size of the dilator must be adapted to the requirements of the individual case, for it is possible to put in such a tight-fitting vaginal plug as to cause sloughing. It should be kept in position by a T bandage. After the glass dilator has been used a week or more, a marked improvement will be apparent. If the cauterization has to be repeated, it should be followed by flaxseed tea and glycerine dressings for several days before using the dilator. The length of time during which the dilator should be worn will depend upon the gravity of the affection. A few weeks may suffice, or it may require months to effect our purpose.

Of course it is essential to study the causes which have led to the local disease; for if the latter is combined with chronic endometritis, displacement of the womb, or if it be due to chlorosis, treatment must be primarily directed to these affections.

It is hardly necessary to add that, while local treatment is in progress, attention should be given to a proper regulation of the diet, tonics administered when required, especially iron in cases of anemia. The patient, if a married woman, should live *absque marito* until a restoration to health occurs.

2. Dr. Whittaker relates an interesting case of a young lady twenty-two years of age, who had never menstruated, although the uterus and mammæ were well developed, and whose general health was excellent, being only interrupted at irregular intervals by headache and abdominal pains. The case was treated with iron, cod-liver oil, quinine, and the entire list of emmenagogues, in conjunction with proper food, exercise, and the most favorable hygienic influences. The foregoing means having failed, the galvanic current was used for three months, one pole being placed over the sacrum and the other over the pubes. Trial was next made of the faradic current, three times a week for four weeks, the sessions being each five minutes in duration. These failed, and six months elapsed before treatment was again instituted. Then the uterus was alternately subjected to galvanization and faradization, after the manner of Golding Bird; one pole tipped with a gilt ball being held at the os uteri, while the other was depressed over the fundus uteri on the abdominal surface. This was continued two months, during the last two weeks of which time the ball electrode was replaced by a sound-shaped pole, which was inserted into the cervix up to the. internal os. Currents of different degrees of intensity, and currents in different directions, were used, but all were alike of no avail. Another interval of four weeks without treatment elapsed. The patient was then induced to allow one more trial. A fine, gold-pointed electrode was inserted directly into the cavity of the uterus, and the other (positive) pole applied over the fundus uteri. An interrupted current from twelve cups was used. The second application was followed by a faint "show." The treatment was faithfully continued for another month without further result, when it was finally discontinued. A short time after (the author does not say how long) the patient presented herself with the joyful intelligence that her menses had appeared and lasted three days. No further history of the case was given.

3. There are a good many ways of cauterizing the uterus, and Dr. Gillespie calls attention to still another expedient, which he characterizes as a "simple, easy and efficient plan for cauterizing the canal of the cervix, and even the cavity of the body." It is as follows:

Take an ordinary sponge tent and coat it with beeswax, and then roll it for some time with a knife in powdered nitrate of silver, which will sink into and adhere to the wax. Then, through a suitable speculum, carry the prepared tent through the cervix, and, if desirable, to the fundus, and let it remain twenty-four hours. The author states that no remedy, in his hands, has done so much good in so short a time, in chronic inflammatory engorgement or ulceration of the os and cervix uteri, and he has never known any unpleasant results from it.

4. In a lecture recently delivered by Dr. Playfair, he stated that chloroform as an anæsthetic during labor has a tendency to do more than is desired; that while it lessens the pain, it at the same time lessens the strength of the uterine contractions, thus prolonging the labor. He likewise objects to its use on the ground of its predisposing to post-partum hemorrhage.

Chloral, on the other hand, he regards as superior in many respects to chloroform, notably in the fact, that while in a marked degree it diminishes their painfulness, it does not seem to lessen the frequency or strength of the pains.

Our author regards it as a great recommendation that chloral is applicable at a period when we would not think of administering chloroform—towards the termination of the first stage of labor, before the complete dilatation of the os, and when the sharp grinding pains produce more suffering, and are less easily borne than the more forcing pains of a later stage.

In a tpye of labor which is very common among women of a highly developed nervous organization, in whom, prior to the rupture of the membranes, and the complete dilatation of the cervix, the pains are very severe, felt chiefly in the back, and productive of little or no effect in dilating the os, he has found chloral especially valuable. In this class of cases it has usually been the custom to administer an opiate in sufficient quantity to produce sleep; but the disadvantage attending this plan is, that the labor is suspended, and much time lost. If, however, chloral be employed instead of the opiate, the same refreshing sleep will be obtained without any suspension of the pains or the protraction of the labor. The character of the contractions, too, will be altered; they will become steady and useful. Also, in cases where there is rigidity and spasm of the cervix, or where the latter is thin and rigid, with a sharp edge, the chloral is exceedingly useful. Soon after it has taken effect, the author has observed, not unfrequently, a thin os, which had remained unaltered in character for many hours, dilate rapidly, far more so than when chloroform is inhaled for this indication.

Dr. Playfair administers fifteen grains of the drug when the first stage of labor is approaching completion; this is repeated in about twenty minutes. Usually, after the second dose, enough has been given to bring the patient under the influence of the remedy. The subsequent administration must be regulated by its effects. If the patient be drowsy and relieved, a third dose need not be given for an hour, and then half the quantity will probably be sufficient to keep the patient in a somnolent condition. It is seldom necessary to give more than a third dose, and the author has never given more than a dram of the medicine in an entire labor. By this lessening the quantity after the second dose, and increasing the intervals between their administration, a full and sufficient effect can be kept up for many hours.

5. In regard to the temperature, pulse and respiration of puerperal women, Dr. Linn has made careful observation of twenty-five cases occurring in the Philadelphia Hospital. The cases were in every respect normal, and furnished the following results: 1. That the normal temperature of the puerperal woman is about 0.6° F. higher than that of the healthy human being, if we accept the statement of Wunderlich, that the mean normal temperature is 98.6° F. 2. That the normal pulse of the puerperal woman is not more frequent than that found under ordinary conditions in a state of perfect health. 3. That the number of respirations is increased, if the statement of physiologists be received, that the number of respirations of the healthy women ranges from 18 to 20 per minute. 4. That the generally received opinion, that the secretion of milk is attended by an increase of temperature of one or more degrees, and an increase in the frequency of the pulse of ten or twelve beats per minute, is erroneous. 5. That a temperature of 100° F., or a pulse of 100 or over per minute, in the lying-in woman, is indicative of some pathological process, which it behooves the accoucheur to discover at once, in order that proper measures may be taken to arrest its development and remove the evil.

